# Supplementary material for: MenT nucleotidyltransferase toxins extend tRNA acceptor stems and can be inhibited by asymmetrical antitoxin binding
Source: Nat Commun. 2023 Aug 17;14:4644. doi: 10.1038/s41467-023-40264-3 (PMC10435456; doi:10.1038/s41467-023-40264-3)
Supplement: Supplementary file 1 — Supplementary Information [file 41467_2023_40264_MOESM1_ESM.pdf]

SUPPLEMENTARY FILE

MenT nucleotidyltransferase toxins extend tRNA acceptor stems and can be inhibited by asymmetrical antitoxin binding

Xibing XU, Ben USHER, Claude GUTIERREZ, Roland BARRIOT, Tom J. ARROWSMITH, Xue HAN, Peter REDDER, Olivier NEYROLLES, Tim R. BLOWER, Pierre GENEVAUX.

This file contains eight Supplementary Figures and one Supplementary Table

Supplementary Fig. S1: Phylogenetic tree analysis of MenA1.

Supplementary Fig. S2: Sequence alignments of MenA1 and MenT1.

Supplementary Fig. S3: MenT1 apo structure contains two protomers.

Supplementary Fig. S4: MenT1 structural alignments.

Supplementary Fig. S5: Overview of tRNA sequencing.

Supplementary Fig. S6: Toxicity and antitoxicity assays with MenT1 and MenA1 mutants.

Supplementary Fig. S7: Detail of MenA1 binding asymmetry.

Supplementary Fig. S8: Alignment of MenT1 and MenAT1 structures.

Supplementary Table S1: Primers and specific sequences used in this work

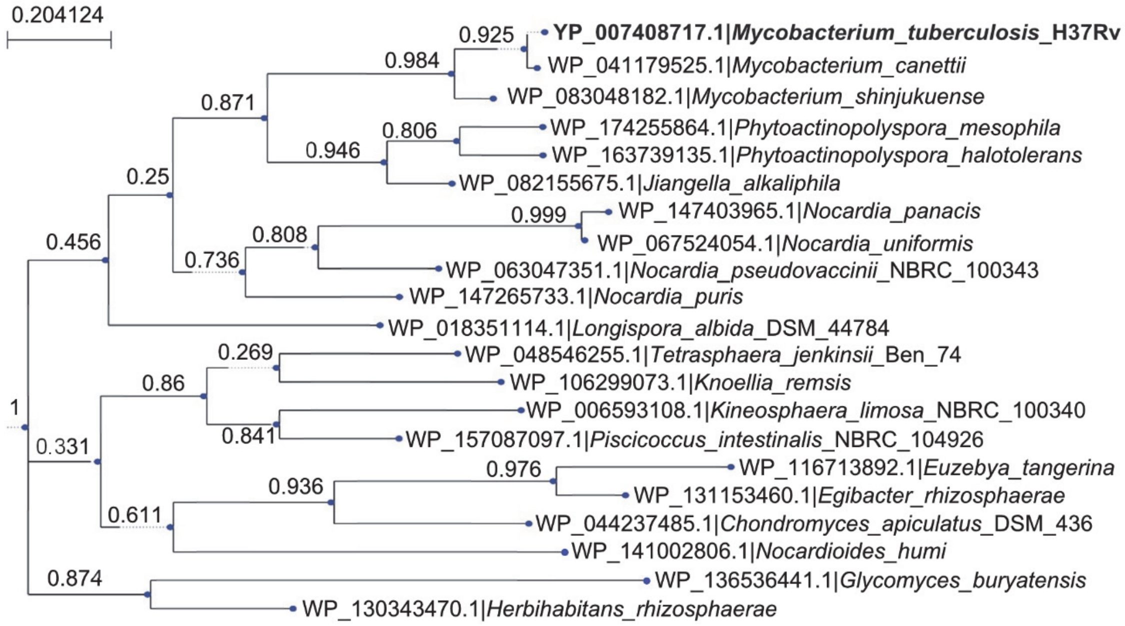

Supplementary Fig. S1: Phylogenetic tree analysis of MenA1. As performed using FlaGs (http://www.webflags.se/).

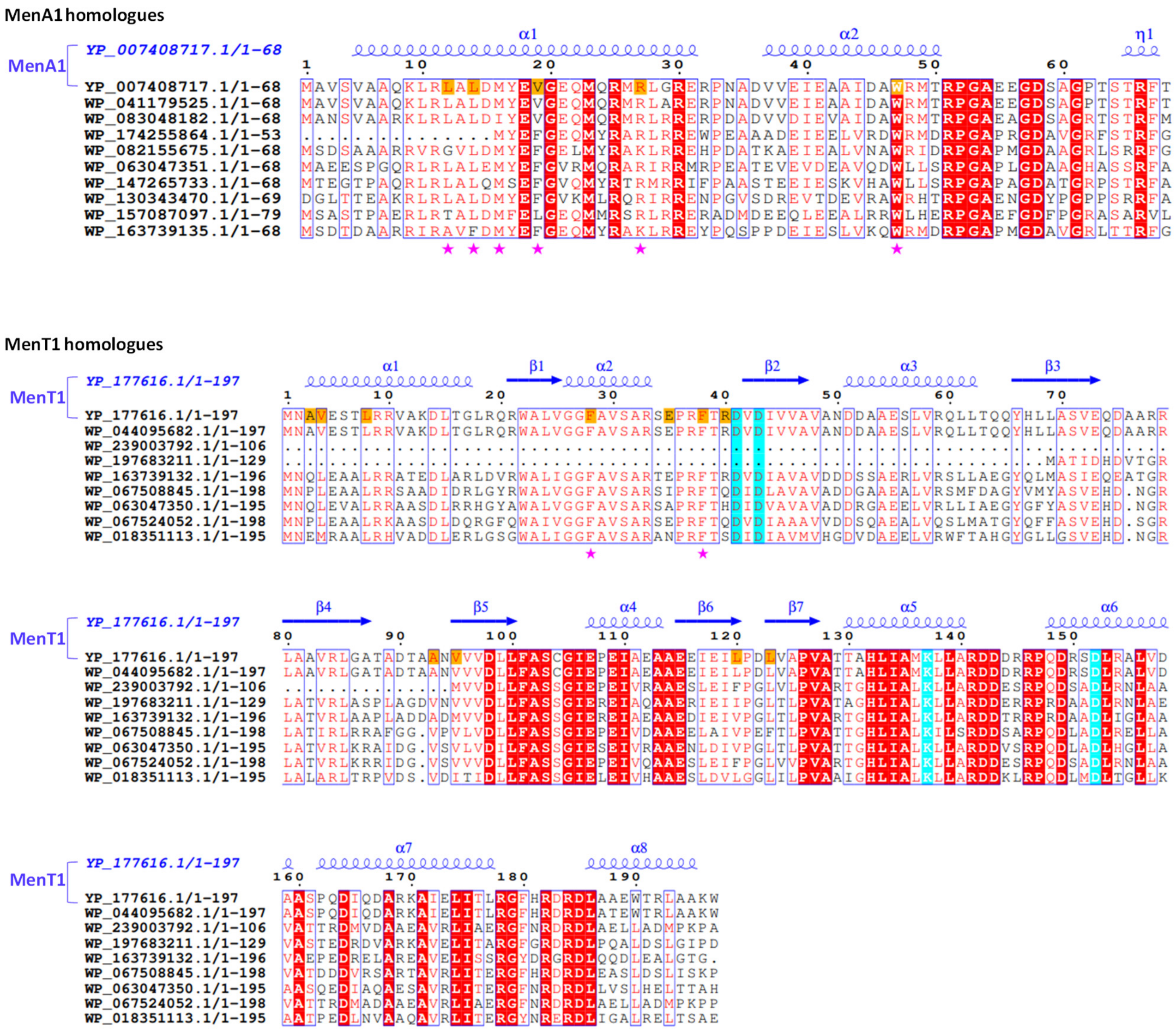

Supplementary Fig. S2: Sequence alignments of MenA1 and MenT1. MenA1 sequences from Fig. 1 were aligned using MUSCLE (https://www.ebi.ac.uk/Tools/msa/muscle/), then formatted in Jalview (https://www.jalview.org/), sorting by pairwise alignment. Residues of interest were then highlighted using ESPript (https://espript.ibcp.fr/ESPript/ESPript/). The shown MenA1 sequences were used to find the paired MenT1 homologues, and the MenT1 sequences were formatted as above. There was no equivalent MenT1 homologue accompanying MenA1 from *Mycobacterium shinjukuense* (WP_083048182) and *Phytoactinopolyspora mesophila* (WP_174255864). Cyan, active site; purple star, substitution mutants; orange, interacting residues in complex.

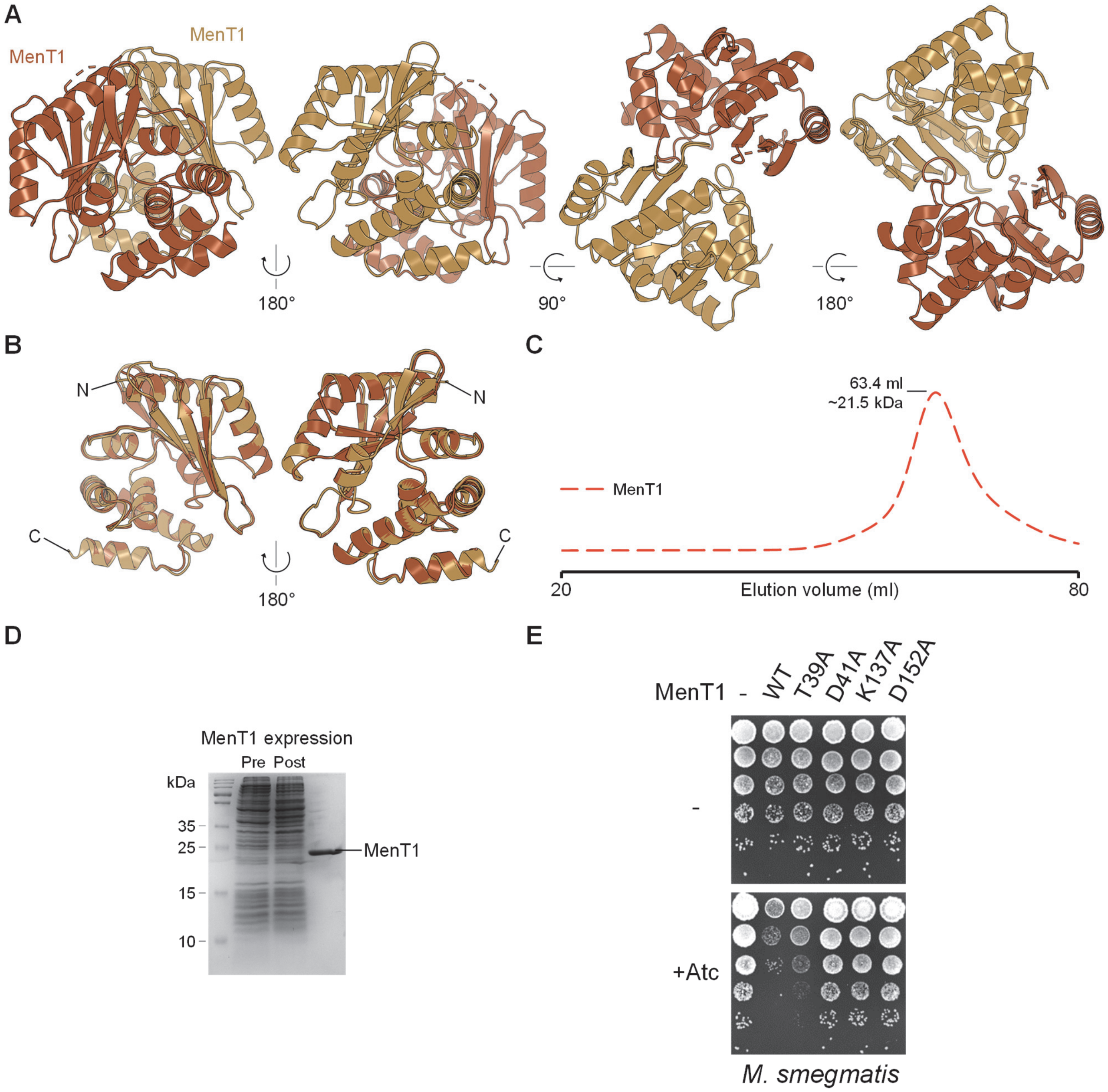

Supplementary Fig. S3: MenT1 apo structure contains two protomers. (**A**) Orthogonal views of the two MenT1 protomers within the asymmetric unit of the crystal structure, shown as cartoons and colored "sand" and "wheat". (**B**) Alignment of the two MenT1 protomers, viewed front and back, and colored as per (**A**). (**C**) Size exclusion chromatogram of expressed and purified MenT1, resolved via a HiPrep 16/60 Sephacryl S-200 SEC column (Cytiva). MenT1 eluted at an elution volume of 63.4 ml, corresponding to a mass equal to its predicted monomeric Mr. (**D**) SDS-PAGE gel of MenT1 samples show expression and purity of the end product. (**E**) *M. smegmatis* transformed with pGMC-vector (-), MenT1 WT or MenT1 T39A, D41A, K137A or D152A mutants were serially diluted and spotted on LB agar plates supplemented with or without Atc inducer (100 ng.ml^{-1}). Plates were incubated for 3 days at 37 °C. Representative results of triplicate experiments are shown.

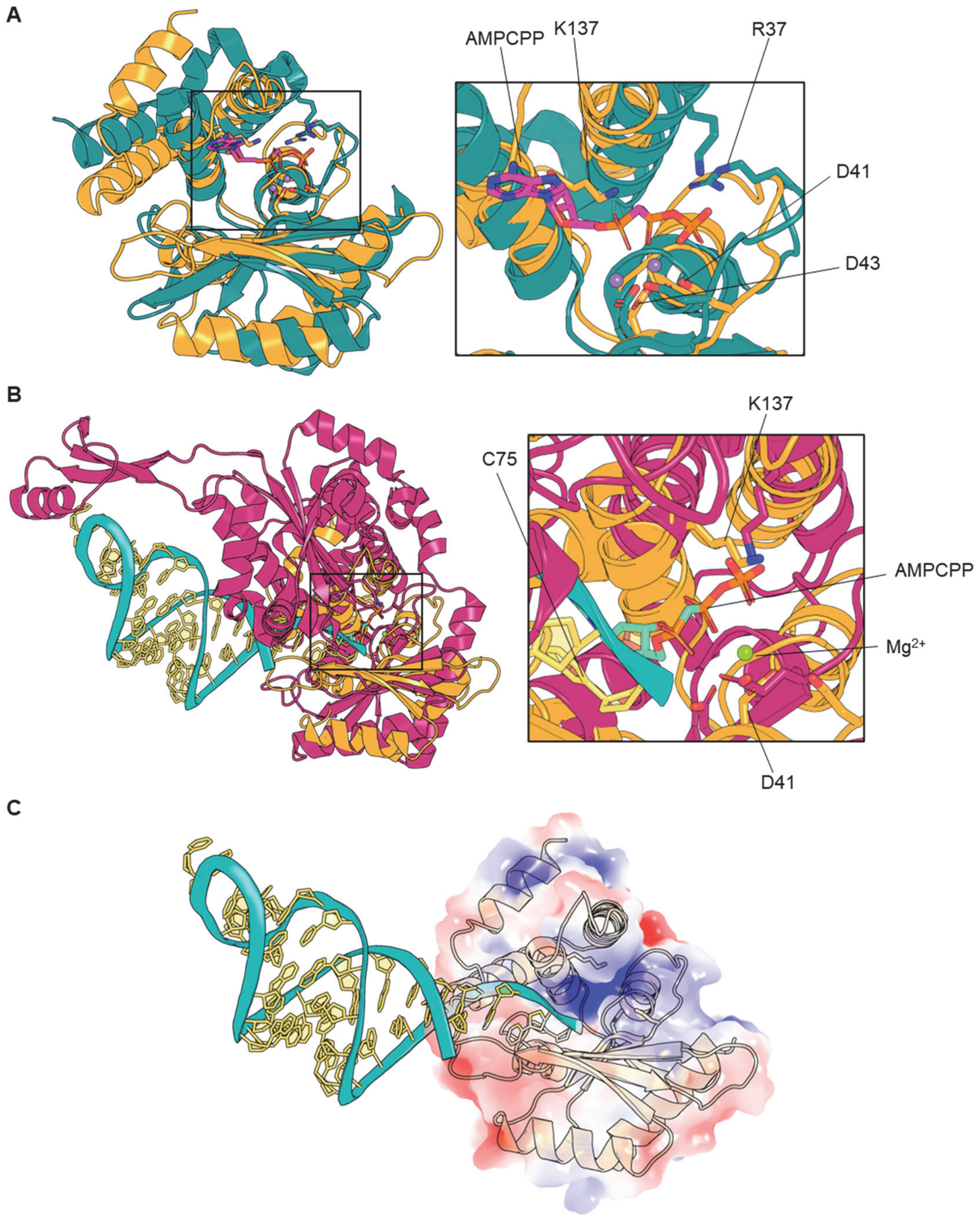

Supplementary Fig. S4: MenT1 structural alignments. (**A**) Structural superposition of $MenT_1$ (gold) and ANT2 (teal, bound to AMPCPP (PDB 4XJE, RMSD = 3.887 Å across 813 atoms)). Triphosphate co-ordinating residues R40, D44, D46, and K147 from ANT2 are structurally conserved in $MenT_1$ (R37, D41, D43, and K137). Conserved aspartates are shown to co-ordinate Mn^{2+} ions (purple spheres). (**B**) Structural superposition of $MenT_1$ (gold) and AMPCPP/tRNA-bound CCA-adding enzyme from *Archaeoglobus fulgidus* (hotpink, PDB 3OVA, RMSD = 10.271 Å across 749 atoms) suggests incoming tRNA C75 base stacks with bound nucleotide substrates, with anchoring of the triphosphate tail mediated by conserved residues as in (**A**). Mg^{2+} ion shown as green sphere. (**C**) Electrostatic surface of $MenT_1$ (generated by APBS PyMol plug-in) positioned with tRNA from (**B**). Blue, electropositive; red, electronegative.

A

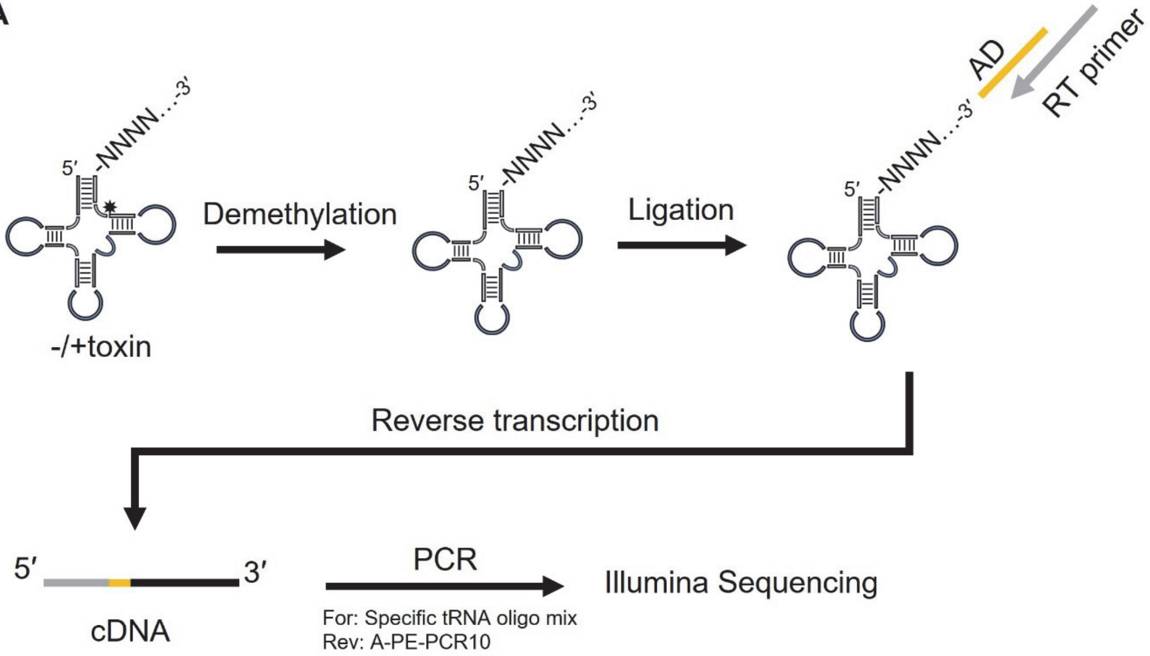

B

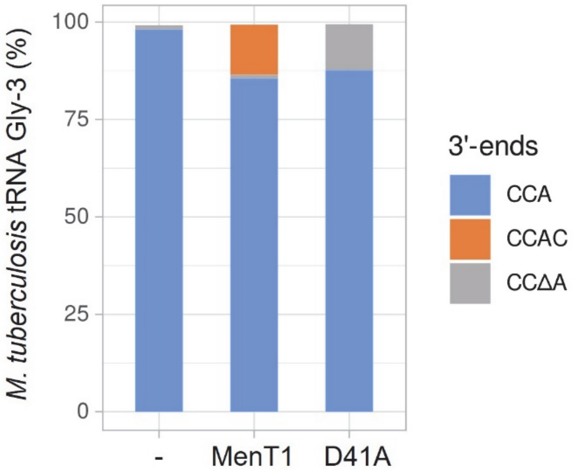

Supplementary Fig. S5: Overview of tRNA sequencing. (**A**) Schematic representation of tRNA 3′ mapping. RNAs are depicted in black, adapter in orange, and the RT primer in grey. (**B**) MenT1 D41A has the ability to remove nucleotide from the tRNA CCA end, as shown in **Fig. 4C**. To confirm this activity, *M. tuberculosis* tRNA Gly-3 was incubated with 5 µM of MenT1 or MenT1 D41A mutant for 1 h at 37 °C in the presence of CTP. The tRNA sequencing method was then performed to confirm the activity of MenT1 D41A on tRNA substrates.

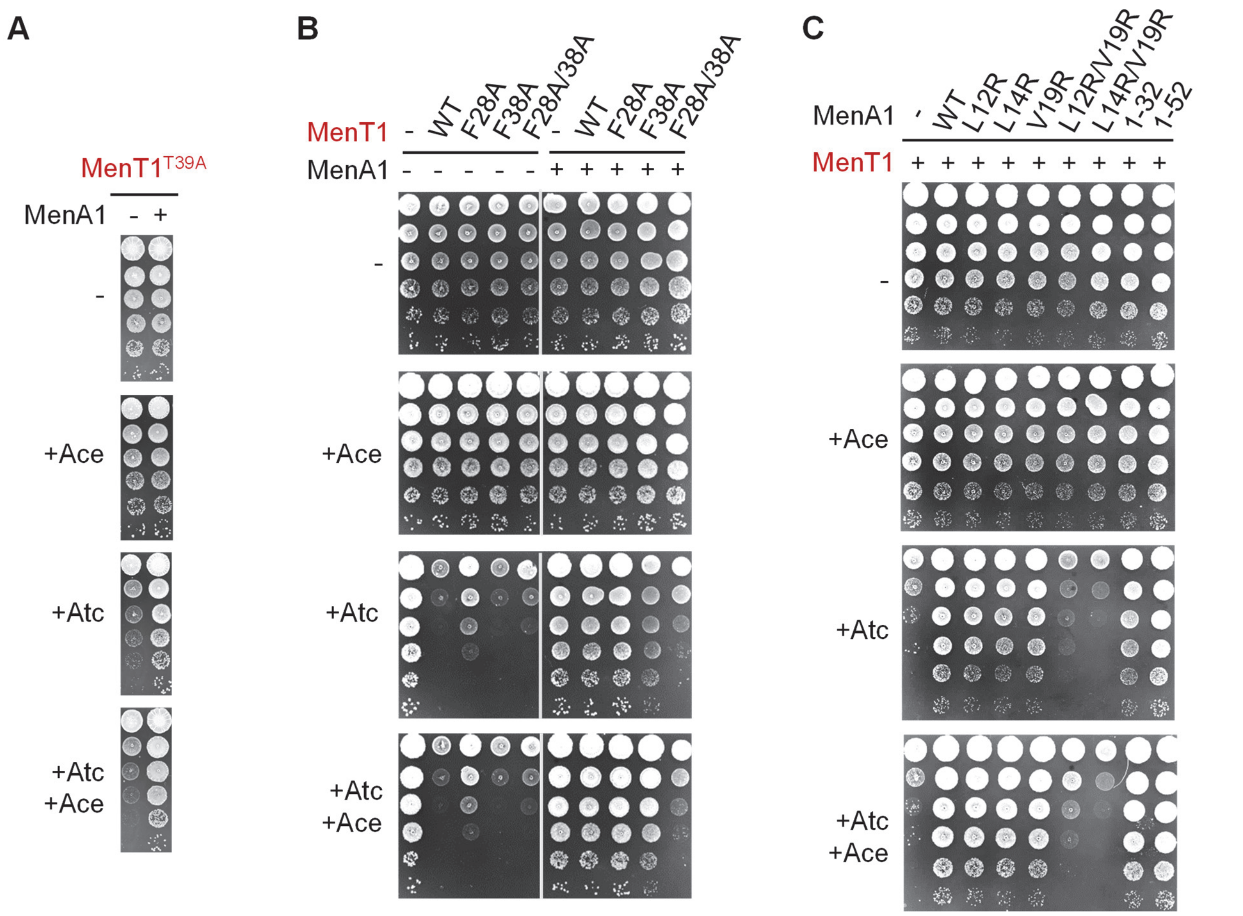

Supplementary Fig. S6: Toxicity and antitoxicity assays with MenT1 and MenA1 mutants. (**A**) MenA1 antitoxin inhibits toxicity of the MenT1 T39A catalytic site substitution *in vivo* in *M. smegmatis*. Co-transformants of *M. smegmatis* containing pGMC-MenT1 T39A and pLAM-vector (-) or pLAM-MenA1 were serially diluted and spotted on LB agar plates in the presence or absence of inducers (Atc, 100 ng.ml^{-1} or Ace, 0.2 %). The plates were incubated for 3 days at 37 °C. (**B** and **C**) Full plating conditions of the growth assays shown in **Fig. 6E**. Representative results of triplicate experiments are shown.

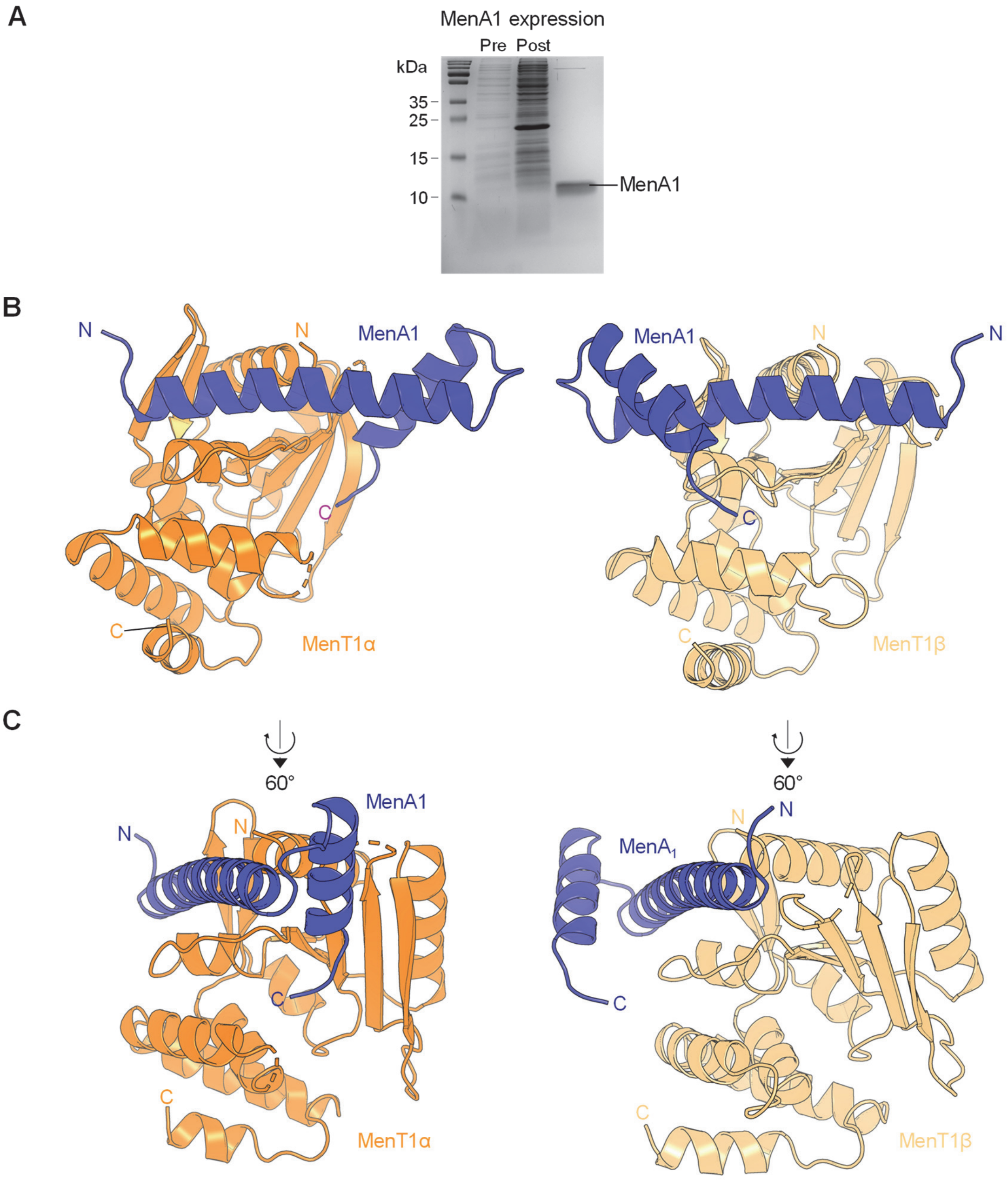

Supplementary Fig. S7: Detail of MenA1 binding asymmetry. (**A**) SDS-PAGE gel of the MenA1 sample shows expression and purity of the end product. (**B** and **C**) Isolated views of MenT1 protomer interactions with MenA1, showing that MenA1 binds on the same face of each MenT1 protomer but in an asymmetric fashion. Proteins colored as per **Fig. 5**. N and C termini are indicated.

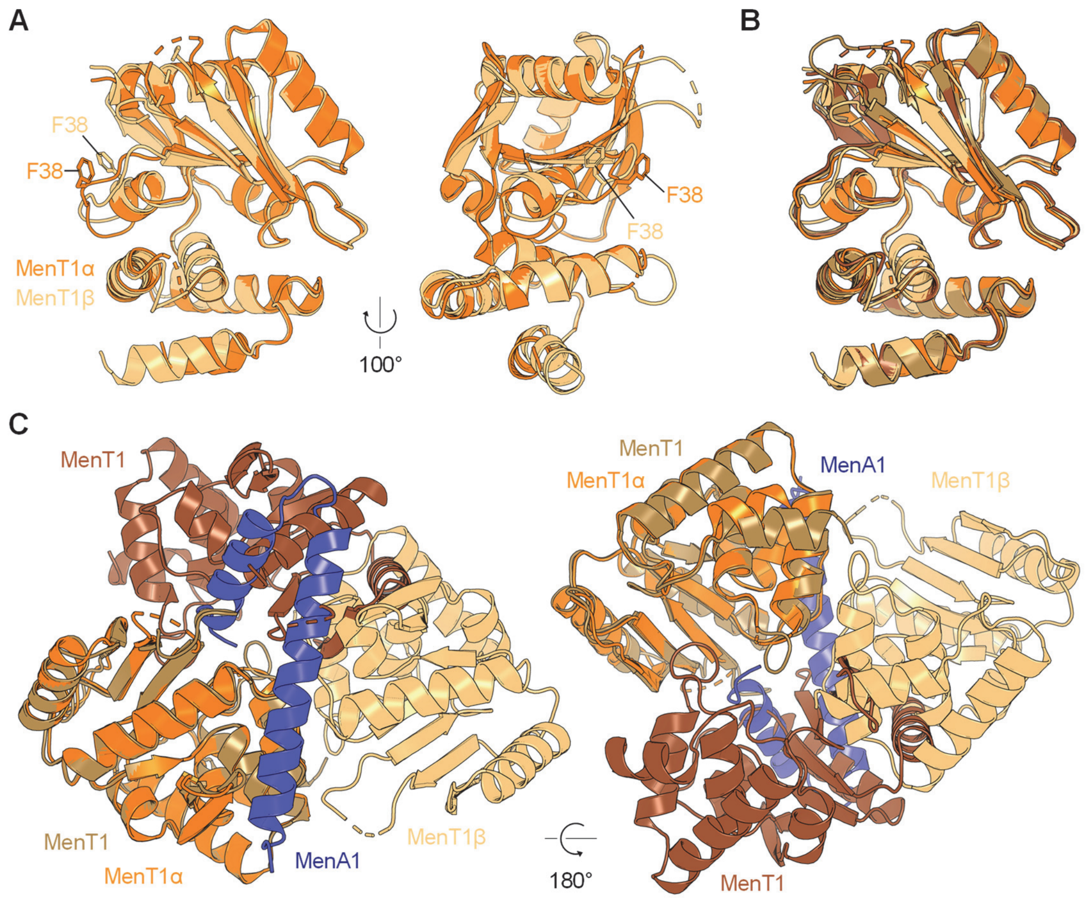

Supplementary Fig. S8: Alignment of MenT1 and MenAT1 structures. (**A**) Alignment of MenT1α and MenT1β from the MenAT1 complex structure, viewed from front and back, shown as cartoons and colored orange (MenT1α) and light orange (MenT1β). (**B**) Alignment of all four MenT1 protomers, shown as cartoons and colored as per **Fig. 5** and **Fig. S2**. (**C**) Alignment of the MenAT1 complex structure and the two protomers from the asymmetric unit of the MenT1 apo structure, aligned through MenT1α and viewed from top and bottom. Proteins are shown as cartoons, colored as per (**A** and **B**), with the addition of MenA1 in blue. The second protomer in the asymmetric unit of MenT1 clashes with the placement of MenA1, demonstrating that the two protomers in the MenT1 apo structure are not positioned for MenA1 binding, and that the presence of two protomers is through crystal packing rather than being biologically significant.

Supplementary Table S1: Primers and specific sequences used in this work

**Gene name**	**Primers**	**Primer sequence (5′ to 3′)**
pK6-MenT1	Rv0078A EcoRI-For	ttgaattccatatgaacgctgtggagtcgacactcc
	Rv0078A HindIII-Rev	ttaagcttggatccttaccacttggcggcgaggc
p29-MenA1	Rv0078B EcoRI-For	ttgaattccatatggcagtttccgtcgctgcgcag
	Rv0078B HindIII-Rev	ttaagcttggatccttatgtgaaccgtgtggacg
pGMC-MenA1T1	Rv0078B In-Fusion-For	gaagacaggctgcccatggcagtttccgtcgctgc
	Rv0078A In-Fusion-Rev	tgtataataaagttgttaccacttggcggcgaggc
pGMC-MenT1	Rv0078A In-Fusion-For	gaagacaggctgcccatgaacgctgtggagtcgac
	Rv0078A In-Fusion-Rev	tgtataataaagttgttaccacttggcggcgaggc
pGMC-MenT1$_{His}$	Rv0078A$_{His}$ In-Fusion-For	gaagacaggctgcccatgcaccaccaccaccaccacagcagcggcaacgctgtggagtcgacact
	Rv0078A In-Fusion-Rev	tgtataataaagttgttaccacttggcggcgaggc
pGMC-MenT1 T39A	Rv0078A T39A For	tccgaaccacgtttcgcccgtgacgtggacatt
	Rv0078A T39A Rev	aatgtccacgtcacgggcgaaacgtggttcgga
pGMC-MenT1 D41A	Rv0078A D41A For	ccacgtttcacccgtgccgtggacattgttgtc
	Rv0078A D41A Rev	gacaacaatgtccacggcacgggtgaaacgtgg
pGMC-MenT1 K137A	Rv0078A K137A For	catctgatcgcgatggcacttcttgcgagagat
	Rv0078A K137A Rev	atctctcgcaagaagtgccatcgcgatcagatg
pGMC-MenT1 D152A	Rv0078A D152A For	ccccaggatcgcagtgctctacgtgcgctcgtg
	Rv0078A D152A Rev	cacgagcgcacgtagagcactgcgatcctgggg
pGMC-MenT1 F28A	Rv0078A F28A For	g cgctggttgg tggtgccgcg gtgtctgcgcga
	Rv0078A F28A Rev	tcgcgcagacac cgcggcacca ccaaccagcg c
pGMC-MenT1 F38A	Rv0078A F38A For	cgatccgaaccacgtgccacccgtgacgtggac
	Rv0078A F38A Rev	gt ccacgtcacgggtggcacgtggttcggatcg
pLAM-MenA1	Rv0078B NdeI-For	ttgaattccatatggcagtttccgtcgctgcgcag

	Rv0078B EcoRI-Rev	ttgaattcttatgtgaaccgtgtggacg
pLAM-MenA1 L12R	Rv0078B L12R For	gcgcagaagctgaggcgcgctctggacatgtac
	Rv0078B L12R Rev	gtacatgtccagagcgcgcctcagcttctgcgc
pLAM-MenA1 L14R	Rv0078B L14R For	aagctgaggctcgctcgggacatgtacgaggtt
	Rv0078B L14R Rev	aacctcgtacatgtcccgagcgagcctcagctt
pLAM-MenA1 V19R	Rv0078B V19R For	ctggacatgtacgagcgtggcgagcagatgcag
	Rv0078B V19R Rev	ctgcatctgctcgccacgctcgtacatgtccag
pLAM-MenA1 N1-52	Rv0078B NdeI-For	ttgaattccatatggcagtttccgtcgctgcgcag
	Rv0078B N1-52 EcoRI-Rev	gagaatccttagggacgcgtcatcctccacg
pLAM- MenA1 N1-32	Rv0078B NdeI-For	ttgaattccatatggcagtttccgtcgctgcgcag
	Rv0078B N1-32 EcoRI-Rev	gagaattcttaccgttcacgacccagcctca
pET-MenA1$_{His}$	Rv0078B$_{His}$ NcoI-For	ttccatggtgcaccaccaccaccaccacagcagcggcgcagtttccgtc gctgcgca
	Rv0078B$_{His}$ BamHI-Rev	ttggatccttatgtgaaccgtgtggacg
pET15b-MenT1$_{His}$ D41A	Rv0078A D41A For	ccacgtttcacccgtgccgtggacattgttgtc
	Rv0078A D41A Rev	gacaacaatgtccacggcacgggtgaaacgtgg
pTRB550	TRB1460 pBAD30-LIC For	tttgaattctttgtttaactttaagaaggagatatatcc
	TRB1462 pBAD30-LIC Rev	tttaagcttggatccctcgaggtcgac
pTRB617-$_{His\text{-}SUMO}$MenA1	TRB1699 Rv0078B For	caacagcagacgggaggtgcagtttccgtcgctgcg
	TRB1700 Rv0078B Rev	gcgagaaccaaggaaaggttattatgtgaaccgtgtgg
pTRB629-$_{His\text{-}Sumo}$MenT1	TRB1701 Rv0078A For	caacagcagacgggaggtaacgctgtggagtcgacac
	TRB1702 Rv0078A Rev	gcgagaaccaaggaaaggttattaccacttggcggcgaggcg
**Construction of H37Rv Δ(*menA1-menT1*)::Zeo^{R}**		
menA1 upstream fragment	MenA1-Up-Fw	gcacgatctgtcgatccagtc
	MenA1-Up-Rv-Zeo	cagtcgatccacgtggagggaaactgccacactcaaagc
menT1 downstream fragment	MenT1-Dw-Fw-Zeo	ccactgagcgtcagacccacgtgctcgctatcgagctgattacc
	MenT1-Dw-Rv	tcgtggagcaagacgtgatg
**tRNA primers**		

Mtb tRNA Ala-1	Mtb tRNA Ala-1 For	attaatacgactcactatagggggctatggcgcagttggtagcgcg
	Mtb tRNA Ala-1 Rev	tggtggagctaaggggattc
Mtb tRNA Ala-2	Mtb tRNA Ala-2 For	attaatacgactcactatagggggctatggcgcagctggtagcgca
	Mtb tRNA Ala-2 Rev	tggtggagctaaggggactc
Mtb tRNA Ala-3	Mtb tRNA Ala-3 For	attaatacgactcactatagggggccttagctcagttggtaga
	Mtb tRNA Ala-3 Rev	tggtggagcctaggggactc
Mtb tRNA Arg-1	Mtb tRNA Arg-1 For	attaatacgactcactataggcgcccgtagctcaacggatagagcatc
	Mtb tRNA Arg-1 Rev	tggcgcgcccgaagagattc
Mtb tRNA Arg-2	Mtb tRNA Arg-2 For	attaatacgactcactataggcccccgtagctcaggggatagagcg
	Mtb tRNA Arg-2/3 Rev	tggtgcccccggcaggattc
Mtb tRNA Arg-3	Mtb tRNA Arg-3 For	attaatacgactcactataggccctcgtagctcaggggatagagcacg
	Mtb tRNA Arg-2/3 Rev	tggtgcccccggcaggattc
Mtb tRNA Arg-4	Mtb tRNA Arg-4 For	attaatacgactcactataggcctccgtagctcaggtgga
	Mtb tRNA Arg-4 Rev	tggtgcccccggcacgactc
Mtb tRNA Asn	Mtb tRNA Asn For	attaatacgactcactatagtcccctgtagctcaattggca
	Mtb tRNA Asn Rev	tggctcccccgggaggactc
Mtb tRNA Asp	Mtb tRNA Asp For	attaatacgactcactatagggccctgtggcgcagttggt
	Mtb tRNA Asp Rev	tggcgaccctgacgggactcg
Mtb tRNA Cys	Mtb tRNA Cys For	attaatacgactcactatagggtggagtggccgagtggtg
	Mtb tRNA Cys Rev	tggaggtggagacgggaatc
Mtb tRNA Gln-1	Mtb tRNA Gln-1 For	attaatacgactcactatagtggggtatggtgtaattggcaa
	Mtb tRNA Gln-1 Rev	tggctggggtaccaggactc
Mtb tRNA Gln-2	Mtb tRNA Gln-2 For	attaatacgactcactatagtccgtcgtggtgtaatcggcag
	Mtb tRNA Gln-2 Rev	tggctccgtcgccaggactc
Mtb tRNA Glu-1	Mtb tRNA Glu-1 For	attaatacgactcactatagggccccgtcgtctagcggccta
	Mtb tRNA Glu-1 Rev	tggtagccccgatgggattc

Mtb tRNA Glu-2	Mtb tRNA Glu-2 For	attaatacgactcactataggccccccttcgtctagacggcct
	Mtb tRNA Glu-2 Rev	tggtaccccctacgggattc
Mtb tRNA Gly-1	Mtb tRNA Gly-1 For	attaatacgactcactataggccgatgtagttcaatggc
	Mtb tRNA Gly-1 Rev	tggagccgatgacgggaatc
Mtb tRNA Gly-2	Mtb tRNA Gly-2 For	attaatacgactcactataggcggatgtagcgcagttggt
	Mtb tRNA Gly-2 Rev	tggagcggatgacgggattc
Mtb tRNA Gly-3	Mtb tRNA Gly-3 For	attaatacgactcactataggcgggcgtagctcaatggt
	Mtb tRNA Gly-3 Rev	tggagcgggcgacgggaatc
Mtb tRNA His	Mtb tRNA His For	attaatacgactcactataggtgagtgtagttcagttggt
	Mtb tRNA His Rev	tggggtgagtgacgggactc
Mtb tRNA Ile	Mtb tRNA Ile For	attaatacgactcactataggggcctatagctcaggcggt
	Mtb tRNA Ile Rev	tggtgggcctaggaggactcgaa
Mtb tRNA Leu-1	Mtb tRNA Leu-1 For	attaatacgactcactataggccctcgtatcccaactggcagagga
	Mtb tRNA Leu-1 Rev	tggtgccctcggtgggattc
Mtb tRNA Leu-2	Mtb tRNA Leu-2 For	attaatacgactcactataggggcgagtggcggaatggcagacgcgctg
	Mtb tRNA Leu-2 Rev	tggtgggcgaagggggactt
Mtb tRNA Leu-3	Mtb tRNA Leu-3 For	attaatacgactcactataggtccgagtggcggaatggcagacgcgcta
	Mtb tRNA Leu-3 Rev	tggtgtccgaggggggactt
Mtb tRNA Leu-4	Mtb tRNA Leu-4 For	attaatacgactcactataggcccccatagcccaattggcagaggc
	Mtb tRNA Leu-4 Rev	tggtgcccccagtcggactc
Mtb tRNA Leu-5	Mtb tRNA Leu-5 For	attaatacgactcactataggcgggcgtgatgaaattggcaa
	Mtb tRNA Leu-5 Rev	tggtgcgggcggagggactc
Mtb tRNA Lys-1	Mtb tRNA Lys-1 For	attaatacgactcactataggcgccgttagctcagttggtagagcag
	Mtb tRNA Lys-1 Rev	tggtgcgccgtcagggtttcgaa
Mtb tRNA Lys-2	Mtb tRNA Lys-2 For	attaatacgactcactataggcccctatagctcagttggtagagcta
	Mtb tRNA Lys-2 Rev	tggtgcccccaccaggactcgaa

Mtb tRNA Met-1	Mtb tRNA Met-1 For	attaatacgactcactatagcgcggggtggagcagctcgg
	Mtb tRNA Met-1 Rev	tggagcggggacaggattcgaac
Mtb tRNA Met-2	Mtb tRNA Met-2 For	attaatacgactcactatagggcgatgtagctcagtcggttagagcga
	Mtb tRNA Met-2 Rev	tggtagcgatggccggactcgaa
Mtb tRNA Met-3	Mtb tRNA Met-3 For	attaatacgactcactatagggggcggtagctcagttggttagagccg
	Mtb tRNA Met-3 Rev	tggtggggcgggcggggctcgaa
Mtb tRNA Phe	Mtb tRNA Phe For	attaatacgactcactatagggccaggtagctcagtcggt
	Mtb tRNA Phe Rev	tggtggccaggggcgggatc
Mtb tRNA Pro-1	Mtb tRNA Pro-1 For	attaatacgactcactatagcggggtgtggcgcagcttggtagcgcg
	Mtb tRNA Pro-1 Rev	tggtcggggtggcgggatttgaa
Mtb tRNA Pro-2	Mtb tRNA Pro-2 For	attaatacgactcactatagcgggctgtggcgcagtttggtagcgcac
	Mtb tRNA Pro-2 Rev	tggtcgggctgacaggatttgaa
Mtb tRNA Pro-3	Mtb tRNA Pro-3 For	attaatacgactcactatagcggggtgtagcgcagcttggtagcgcat
	Mtb tRNA Pro-3 Rev	tggtcggggtgacaggattt
Mtb tRNA Ser-1	Mtb tRNA Ser-1 For	attaatacgactcactatagggtggcgtgtccgagcggcctaag
	Mtb tRNA Ser-1/4 Rev	tggcggtggcggagggattt
Mtb tRNA Ser-2	Mtb tRNA Ser-2 For	attaatacgactcactatagggaggcgtgccagagcggccga
	Mtb tRNA Ser-2 Rev	tggcggaggcgagaggattt
Mtb tRNA Ser-3	Mtb tRNA Ser-3 For	attaatacgactcactatagggaggattcgcctagtggcctat
	Mtb tRNA Ser-3 Rev	tggcggaggatgcgggattt
Mtb tRNA Ser-4	Mtb tRNA Ser-4 For	attaatacgactcactatagggtggcgtggcagagcggcctaat
	Mtb tRNA Ser-1/4 Rev	tggcggtggcggagggattt
Mtb tRNA Thr-1	Mtb tRNA Thr-1 For	attaatacgactcactataggccgccttagctcagtcggt
	Mtb tRNA Thr-1 Rev	tggagccgcctaggagaatc
Mtb tRNA Thr-2	Mtb tRNA Thr-2 For	attaatacgactcactataggccccttagctcagtcggc

	Mtb tRNA Thr-2 Rev	tggagccccctaacggaatc
Mtb tRNA Thr-3	Mtb tRNA Thr-3 For	attaatacgactcactataggcctccttagctcagtggta
	Mtb tRNA Thr-3 Rev	tggagccccctgtcaggatt
Mtb tRNA Trp	Mtb tRNA Trp For	attaatacgactcactatagaggggcgtagctcaactggc
	Mtb tRNA Trp Rev	tggcaggggcgacaggacttgaa
Mtb tRNA Tyr	Mtb tRNA Tyr For	attaatacgactcactatagggcaggttgcccgagcggcc
	Mtb tRNA Tyr Rev	tggtggcaggtgcaggattc
Mtb tRNA Val-1	Mtb tRNA Val-1 For	attaatacgactcactatagggtcccgtggctcagtgggagagcgt
	Mtb tRNA Val-1 Rev	tggtggtcccggctgggatc
Mtb tRNA Val-2	Mtb tRNA Val-2 For	attaatacgactcactataggcgcgattagctcagcgggagagcgc
	Mtb tRNA Val-2 Rev	tggtgcgcgatactgggatt
Mtb tRNA Val-3	Mtb tRNA Val-3 For	attaatacgactcactataggggcgcgtagctcagcggt
	Mtb tRNA Val-3 Rev	tggtgggcgcggacgggatc
T7-tRNA	T7-For	attaatacgactcactat
	HDV-Rev	aaacgacggccagtgccaag
	HDV short-Rev	cttctcccttagcctaccg
Mtb tRNA T7-Gly-3-HDV	T7-Gly3-HDV In-Fusion-For	gaagacaggctgcccattaatacgactcactata
	T7-Gly3-HDV In-Fusion-Rev	tgtataataaagttgaaacgacggccagtgccaa
	pGMC-Gly3GCAF	gcccgctgcagggtcggcatggcatctcc
	pGMC-Gly3GCAR	gaccctgcagcgggcgacgggaatcg
	pGMC-Gly3TCAF	gcccgcttcagggtcggcatggcatctcc
	pGMC-Gly3TCAR	gaccctgaagcgggcgacgggaatcg
	pGMC-Gly3ACAF	gcccgctacagggtcggcatggcatctcc
	pGMC-gly3ACAR	gaccctgtagcgggcgacgggaatcg
	pGMC-gly3GCAF	ttcccgtcgcccgctgcagggtcggcatggcat
	pGMC-gly3GCAR	atgccatgccgaccctgcagcgggcgacgggaa
	pGMC-gly3TCAR	atgccatgccgaccctgaagcgggcgacgggaa
	pGMC-gly3TCAF	ttcccgtcgcccgcttcagggtcggcatggcat
	pGMC-gly3ACAF	ttcccgtcgcccgctacagggtcggcatggcat
	pGMC-gly3ACAR	atgccatgccgaccctgtagcgggcgacgggaa
	Gly3-HDV For	cgattcccgtcgcccgctccagggtcggcatggcatctc
	Gly3-HDV Rev	gagatgccatgccgaccctggagcgggcgacgggaatcg
	T7-Gly3 For	attaatacgactcactatagcgggcgtagctcaatggta
	HDV-Rev	aaacgacggccagtgccaag
	Gly3mtbFor	gcgggcgtagctcaatggtagagccctagtcttccaaactagcgacgcg ggttcgattcccgtcgcccgctcca

	HDVFor	gggtcggcatggcatctccacctcctcgcggtccgacctgggctacttcg gtaggctaagggagaagcttggcactggccgtcgttt
**Sequences of T7-tRNA-HDV constructs**		
T7-His-HDV<br>attaatacgactcactatagtgagtgtagttcagttggtagagcaccaggttgtgatcctgggtgtcgcgggttcgagtcccgtcactcaccc cagggtcggcatggcatctccacctcctcgcggtccgacctgggctacttcggtaggctaagggagaagcttggcactggccgtcgttt		
T7-Leu-3-HDV<br>attaatacgactcactatagtccgagtggcggaatggcagacgcgctagcttgaggtgctagtgccctactaatgggcgtgggggttcaa gtccccctcggacaccagggtcggcatggcatctccacctcctcgcggtccgacctgggctacttcggtaggctaagggagaag		
T7-Met-2-HDV<br>attaatacgactcactataggcgatgtagctcagtcggttagagcgaacgactcataatcgttaggtcgccggttcgagtccggccatcgct accagggtcggcatggcatctccacctcctcgcggtccgacctgggctacttcggtaggctaagggagaagcttggcactggccgtcgtt t		
T7-Ser-4-HDV<br>attaatacgactcactataggtggcgtggcagagcggcctaatgcactcgccttgaaagcgagagacggctaacaccgtccgggggttc aaatccctccgccaccgccagggtcggcatggcatctccacctcctcgcggtccgacctgggctacttcggtaggctaagggagaag		
T7-Gly-3-HDV<br>attaatacgactcactatagcgggcgtagctcaatggtagagccctagtcttccaaactagcgacgcgggttcgattcccgtcgcccgctc cagggtcggcatggcatctccacctcctcgcggtccgacctgggctacttcggtaggctaagggagaagcttggcactggccgtcgttt		
**RNA Seq primers**		
Adaptor	3p-v4	Phosphate-***gtatct****nnnnnnnnnnnnnnnTGAG*cctcggttggtgccg-/SpacerC3/
Barcode	D6E	ctctttccctacacgacgctcttccgatctnacaccggcaccaaccgagg
	D6F	ctctttccctacacgacgctcttccgatctnggtacggcaccaaccgagg
	D6O	ctctttccctacacgacgctcttccgatctnattgcggcaccaaccgagg
	D6P	ctctttccctacacgacgctcttccgatctngacgcggcaccaaccgagg
	D6Q	ctctttccctacacgacgctcttccgatctntgttcggcaccaaccgagg
	D6A	ctctttccctacacgacgctcttccgatctntacacggcaccaaccgagg
	D6B	ctctttccctacacgacgctcttccgatctngtatcggcaccaaccgagg
	D6C	ctctttccctacacgacgctcttccgatctncgtccggcaccaaccgagg
PCR	EMOTE-B_i7RPI7_GATCTG	caagcagaagacggcatacgagatgatctggtgactggagttcagacgtgtgc
	EMOTE-B_i7RPI1_CGTGAT	caagcagaagacggcatacgagatcgtgatgtgactggagttcagacgtgtgc
	**tRNAs oligoFor mix**	
	Ala tRNA seqFw	ctggagttcagacgtgtgctcttccgatct ggggctatggcgcagttggta
	Asn- Asp- Phe-Trp tRNA seqFw	ctggagttcagacgtgtgctcttccgatct gtagctcaattggcaagagc
	Cys tRNA seqFw	ctggagttcagacgtgtgctcttccgatct gtggccgagtggtgaggca
	Gln tRNA seqFw	ctggagttcagacgtgtgctcttccgatctaattggcaacacagctgatt
	Glu tRNA seqFw	ctggagttcagacgtgtgctcttccgatctcccccatcgtctagtggcct
	LeuCAG GAG TAG tRNA seqFw	ctggagttcagacgtgtgctcttccgatctgtggcggaatggcagacgcg
	LeuCAA TAA tRNA seqFw	ctggagttcagacgtgtgctcttccgatctcgtatcccaactggcagagg
	Lys tRNA seqFw	ctggagttcagacgtgtgctcttccgatctgttagctcagttggtagagc

	Met tRNA seqFw	ctggagttcagacgtgtgctcttccgatctgttagatcggcgggctcata
	Pro tRNA seqFw	ctggagttcagacgtgtgctcttccgatctcgggctgtggcgcagcttgg
	Sec tRNA seqFw	ctggagttcagacgtgtgctcttccgatctggaggcgtatccggtctggt
	SerGGA CGA TGA tRNA seqFw	ctggagttcagacgtgtgctcttccgatctagtggcctaaggcgctcgcct
	SerGCT Tyr tRNA seqFw	ctggagttcagacgtgtgctcttccgatctggaggcttgcccgagcggcc
	Thr tRNA seqFw	ctggagttcagacgtgtgctcttccgatctcttagctcagtcggcagagc
	Val tRNA seqFw	ctggagttcagacgtgtgctcttccgatctcgagtagctcagcgggagag c
	Arg tRNA seqFw	ctggagttcagacgtgtgctcttccgatctgcccccgtagctcaggggat
	Gly His tRNAs seqFw	ctggagttcagacgtgtgctcttccgatctgcgggcgtagctcaatggta
	Ile tRNAs seqFw	ctggagttcagacgtgtgctcttccgatctgggcctatagctcaggcggt
	A-PE-PCR10	aatgatacggcgaccaccgagatctacactctttccctacacgacg

RNA-Seq read structure

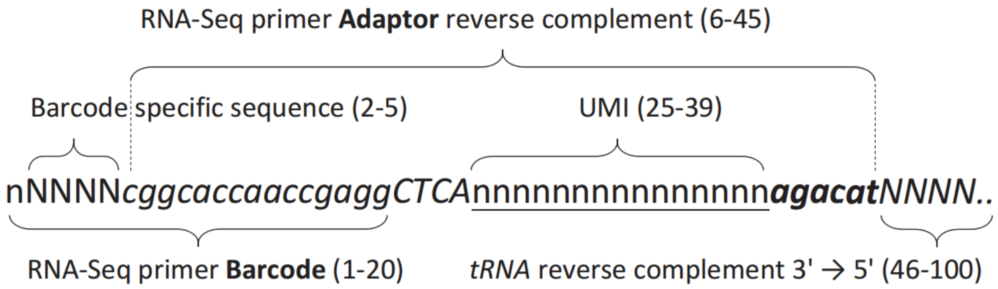

Position 1: random nucleotide

Position 2-5: RNA-Seq Barcode specific sequence from primer D6E, D6F, D6O, ..., in capital in the Barcode sequences

Position 6-24: sequence resulting from RNA-Seq Adaptor ligation i.e., end of Barcode (in italic in the Barcode sequence) followed by CTAC, the reverse complement of Adaptor (TGAG in capital in the Adaptor sequence)

Position 25-39: Unique Molecule Identifier (UMI) for PCR duplicate removal, underlined in the Adaptor sequence

Position 40-45: end of Adaptor reverse complement, agacat, in the RNA-Seq read (gtatct beginning of Adaptor in bold)

Position 46-100: reverse complement of the tRNA 3'end sequence
