# Supplementary material for: MenT nucleotidyltransferase toxins extend tRNA acceptor stems and can be inhibited by asymmetrical antitoxin binding
Source: Nat Commun. 2023 Aug 17;14:4644. doi: 10.1038/s41467-023-40264-3 (PMC10435456; doi:10.1038/s41467-023-40264-3)
Supplement: Supplementary file 3 — Description of additional supplementary files [file 41467_2023_40264_MOESM3_ESM.pdf]

Description of additional supplementary files

Supplementary Datasheet 1: tRNA sequencing data and FlaGs gene information.
